# New Treatments for Acromegaly in Development

**DOI:** 10.1210/clinem/dgad568

**Published:** 2023-09-27

**Authors:** Mônica R Gadelha, Ana Carolina Gadelha, Leandro Kasuki

**Affiliations:** Endocrine Unit and Neuroendocrinology Research Center, Medical School and Hospital Universitário Clementino Fraga Filho—Universidade Federal do Rio de Janeiro, Rio de Janeiro 21941-913, Brazil; Neuroendocrine Unit—Instituto Estadual do Cérebro Paulo Niemeyer, Secretaria Estadual de Saúde, Rio de Janeiro 20231-092, Brazil; Neuropathology and Molecular Genetics Laboratory, Instituto Estadual do Cérebro Paulo Niemeyer, Secretaria Estadual de Saúde, Rio de Janeiro 20231-092, Brazil; Endocrine Unit and Neuroendocrinology Research Center, Medical School and Hospital Universitário Clementino Fraga Filho—Universidade Federal do Rio de Janeiro, Rio de Janeiro 21941-913, Brazil; Endocrine Unit and Neuroendocrinology Research Center, Medical School and Hospital Universitário Clementino Fraga Filho—Universidade Federal do Rio de Janeiro, Rio de Janeiro 21941-913, Brazil; Neuroendocrine Unit—Instituto Estadual do Cérebro Paulo Niemeyer, Secretaria Estadual de Saúde, Rio de Janeiro 20231-092, Brazil

**Keywords:** acromegaly, new drugs, somatostatin receptor ligands, paltusotine, octreotide SC depot, GH receptor antagonist

## Abstract

Acromegaly treatment has greatly evolved in recent decades, but there are still patients whose acromegaly is not controlled with currently available treatments, and there is a need to improve the treatment burden. Fortunately, there are new treatments under development that may increase treatment efficacy and convenience.

Adequate treatment of acromegaly is essential because achieving normalization of growth hormone (GH) and insulin-like growth factor type I (IGF-I) levels leads to an important reduction in morbidity and normalization of mortality rates (1). Surgery is the first-line treatment in the majority of patients, and medical therapy is indicated in patients not controlled with surgery or in those for whom there is a contraindication to surgical treatment (2). Despite the availability of many drugs that act in the GH-secreting adenomas (cabergoline, lanreotide, octreotide, and pasireotide) and of a GH receptor antagonist (pegvisomant), there are still patients who do not achieve disease control (3). In addition, except for cabergoline, the currently available drugs are injectable drugs, which generates a burden on the treatment (4). Therefore, the development of new treatments may address unmet needs in the treatment of acromegaly.

Acromegaly management is one of the main research lines of our group, and we have participated in several clinical trials (5-9). Additionally, we have great interest in the new developments in this area and follow the advancements in the field (10-13). Unfortunately, some promising drugs in phase 1 and/or 2 of clinical development have not been successful in later stages (10, 11), but there are other drugs under development with potential for future use in clinical practice (11). In this review, we will discuss new drugs that are under development for the treatment of acromegaly.

## Search Strategy

We performed a PubMed database search for articles in the English language on the following search terms: “new drugs AND acromegaly” and “new treatments AND acromegaly.” Selected papers cited in the identified articles were also considered. We also searched congress abstracts and ClinicalTrials.gov for studies with new treatments for acromegaly.

## Somatotropinoma-Directed Drugs

Currently, 2 drug classes act directly in GH-secreting adenoma: somatostatin receptor ligands (SRLs) (lanreotide and octreotide [first generation] and pasireotide [second generation]) and dopamine agonists (only cabergoline is used to treat acromegaly) (2).

### Paltusotine

Paltusotine (formerly known as CRN00808) is a first in class, once daily oral nonpeptide selective agonist of somatostatin receptor type 2 (SST2), which is the most expressed receptor in somatotropinomas together with somatostatin receptor type 5 (SST5) (14, 15). Paltusotine has >4000-fold selectivity for SST2 compared with any of the other somatostatin receptor (SST) subtypes and is a highly potent agonist of SST2 (median effective concentration = 0.25 nM) (14). Paltusotine is a small molecule that intrinsically permeates the intestinal epithelium without the need for absorption enhancers to allow for oral administration. The oral solution of paltusotine has been shown to have 70% bioavailability in humans (16).

A randomized, double-blind, placebo-controlled, single-center, single and multiple ascending dose phase 1 study was conducted in healthy male volunteers The single-dose cohorts (n = 41 active, n = 14 placebo) received oral paltusotine 1.25, 2.5, 5, 10, and 20 mg (solution) and 40 and 60 mg (capsules) (17). The multiple-dose cohorts (n = 24 active, n = 12 placebo) received oral paltusotine capsules once daily in increasing doses (5 mg dose for 7 days and doses of 10, 20, and 30 mg daily for 10 days). The main outcomes were pharmacokinetics, pharmacodynamics (alterations in GH-releasing hormone stimulated GH and IGF-I), safety, and tolerability.

In single-dose cohorts receiving the oral solution while fasting, paltusotine reached a median peak concentration at 1.3 to 2.2 hours, showing rapid absorption, followed by terminal elimination at 22 to 29 hours (17). Dose-dependent suppression of GH-releasing hormone–stimulated GH secretion was observed, with maximal inhibition being observed at the 10-mg dose (91% suppression). IGF-I levels after a single dose (1.25-20 mg) of paltusotine showed no significant change compared with day −1 or placebo. A small loss of relative bioavailability with the capsule compared with the solution was observed, which is expected due to the time necessary for disintegration and dissolution of a capsule. In the multiple-dose cohorts, the drug achieved steady state in 3 to 5 days following once-daily dosing, with near-maximal suppression of IGF-I levels (approximately 35%) being observed after approximately 7 days (17).

Paltusotine was well tolerated, with the most common side effects in single-dose paltusotine–treated participants being headaches (n = 5) and in multiple-dose cohorts being diarrhea (n = 4), abdominal pain (n = 4), and headache (n = 3) (17). Treatment-emergent adverse events (AEs) occurred mainly at a dose level of 10 mg or higher (78% of the events in multiple-dose cohorts). Two volunteers experienced elevation of amylase or lipase, and 1 discontinued the drug after 5 days of the 20-mg dose due to elevations of 2.0 and 3.9 times the upper limit of normal (ULN) of amylase and lipase, respectively. Amylase and lipase levels returned to normal after study drug discontinuation, and there were no clinical consequences associated with this event. In the other subject, there was an increase of 1.7 times the ULN of lipase with normal amylase levels after the tenth (last) dose of 30 mg, with spontaneous resolution after stopping the drug.

Elevation of amylase and lipase was observed at a frequency similar to other SRLs (18). No life-threatening AEs or death occurred.

After the phase 1 study, ACROBAT Edge, an open-label, prospective, multicenter, nonrandomized, single-arm, phase 2 study, was performed (5). The dose range evaluated in phase 2 initiated at 10 mg per day, a dose that resulted in maximal IGF-I suppression in healthy volunteers. The dose range evaluated was capped at 40 mg per day as it was also noted in the phase 1 study that doses of the capsule formulation above this level did not result in increased drug exposure. Patients with uncontrolled acromegaly using long-acting release (LAR) or lanreotide injection monotherapy in stable doses (Group 1; n = 25) constituted the primary analysis cohort. Exploratory groups were also enrolled in the study including those who used first-generation SRLs in association with cabergoline (without disease control [Group 2; n = 10] or with disease control [Group 3; n = 5]); pasireotide LAR in monotherapy with disease control (Group 4; n = 4); and pegvisomant in monotherapy with disease control (Group 5; n = 3).

The primary analysis cohort consisted of 25 patients who started paltusotine 10 mg daily 4 weeks after the last injectable SRL dose, followed by protocol-defined 10-mg dose increments to a maximum dose of 40 mg/day at weeks 4, 7, and 10 according to IGF-I levels (5). Patients were treated for a total of 13 weeks, and a washout period of 4 weeks was performed. The primary efficacy endpoint was the change in IGF-I levels from baseline to week 13. There was no significant change in GH and IGF-I levels from baseline (median GH and IGF-I levels of 0.69 ng/mL and 1.34 × ULN, respectively) to week 13 (median change of −0.05 ng/mL and −0.03 × ULN, respectively), showing that the biochemical response to injectable first-generation SRLs was maintained after switching to paltusotine. In 18 patients, the dose was uptitrated to 40 mg/day.

After the washout period, there was an increase of 109% and 39% in GH and IGF-I levels, respectively. Patient-reported outcomes were evaluated using a daily Acromegaly Symptom Diary (developed based on FDA guidance) and a Patient Global Impression of Improvement (19). As expected in a study designed to maintain baseline levels of disease control, there was no change in the Acromegaly Symptom Diary, and at the end of the treatment period, according to the Patient Global Impression of Improvement, no patient reported any degree of worsening, while 11 (23.4%) reported that they had “very much improved” or “much improved,” whereas 26 (55.3%) had “minimally improved” or noted no change (5).

Patients in all 5 groups were included in the safety and tolerability analysis. Paltusotine was well tolerated, with no serious AEs related to the drug or drug discontinuation due to side effects (5). The most common AEs (occurring in >10% of patients) were arthralgia, diarrhea, fatigue, headache, hyperhidrosis, paresthesia, and peripheral swelling.

Another phase 2 trial that evaluated patients with normal IGF-I at baseline on monotherapy with octreotide or lanreotide (ACROBAT Evolve) stopped enrollment in April 2020 based on positive Edge results (10).

The ACROBAT Advance study is an open-label, single-arm extension study that included patients from both ACROBAT phase 2 Edge and Evolve studies and was planned to analyze long-term treatment with paltusotine (up to 4 years) (20). In this trial, adjunctive treatment with cabergoline or pegvisomant was allowed in patients who had not achieved disease control (normal age-adjusted IGF-I levels) with a maximum dose of paltusotine (40 mg) (20). Recently, an interim analysis of 2 years of treatment that included 43 patients (32 from Edge and 11 from Evolve) showed that IGF-I levels remained stable for up to 103 weeks (1.15 × ULN at baseline, 1.08 × ULN at week 51, 1.01 × ULN at week 77, and 1.10 × ULN at week 103) (21). The safety profile was similar to that in previous studies, and no serious AEs were reported.

More recently, a second-generation, spray-dried dispersion tablet formulation of paltusotine was developed to allow for higher dose options and a reduced postdose fasting requirement relative to the capsule formulation used in the phase 1 and 2 studies (22). The fasting requirement is based on decreased drug absorption when administered with a high-fat meal documented in the original phase 1 study (17). The tablet formulation is currently being used in 2 ongoing phase 3 studies to assess the safety and efficacy of paltusotine (PATHFNDR-1 and 2; clinicaltrilas.gov registration numbers NCT04837040 and NCT05192382, respectively). Phase 3 trial participants are currently asked to take paltusotine in the morning after at least a 6-hour overnight fast and then wait 1 hour before eating.

PATHFNDR-1 is a multicenter, randomized, double-blind, placebo-controlled trial designed to evaluate safety and efficacy of paltusotine in patients with controlled acromegaly (IGF-I < 1 × ULN) on first-generation SRL injections. The primary endpoint is the proportion of patients maintaining IGF-I levels at ≤1.0 times the ULN on paltusotine compared with placebo. PATHFNDR-1 enrolled 58 patients, and top-line results are expected in September 2023.

PATHFNDR-2 is another placebo-controlled clinical trial designed to evaluate the safety and efficacy of paltusotine in patients with acromegaly who are treatment-naïve or not currently receiving medical therapy. The primary endpoint is the proportion of patients on paltusotine who achieve IGF-I levels ≤1.0 times the ULN at the end of the randomized control phase compared with placebo. The study recently completed enrollment of 112 participants.

### Octreotide Subcutaneous Depot (CAM2029)

CAM2029 consists of a new formulation of injectable subcutaneous octreotide based on the FluidCrystal injection depot technology (23, 24). This technology comprises a lipid drug delivery system that, when exposed to aqueous environments, spontaneously forms liquid crystal nanostructures. The lipid composition and liquid crystal nanostructure determine the release rate of the drug, being able to provide a controlled and high drug exposure. Its advantages include the following: requirement of only monthly subcutaneous (SC) administration; use of thin needles; compatibility with prefilled administration devices such as prefilled syringes and prefilled pens enabling patient self-administration; drug stability at room temperature; and 500% increased plasma exposure when compared with octreotide LAR (25).

A phase 1, randomized, open label, repeated dose, active control, parallel study was performed to analyze the effects in 122 healthy volunteers randomly assigned to 8 different groups (25). On Day 0, all participants received a single dose of octreotide immediate release. Blood samples were collected for the pharmacokinetics profile of octreotide immediate release. The results showed a rapid increase in peak concentrations of the drug and a progressive reduction in the 24 hours after the injection. After a 7-day washout period, each of the groups received 3 repeated once monthly doses on Days 7, 35, and 63 of the study: CAM2029 A 10, 20, or 30 mg, CAM2029 B 30 mg, CAM2029 C 10, 20, or 30 mg and 30 mg of octreotide LAR. CAM2029 A, B, and C differ regarding the cosolvent levels. Pharmacokinetics and pharmacodynamics profiles of both drugs were evaluated. The results revealed a dose-dependent increase in CAM2029 A and C concentrations in the plasma. Octreotide SC depot B peaks were observed immediately after drug administration and were approximately 50 times higher than the octreotide LAR initial peak; later, its concentration progressively decreased.

A 4- to 5-fold greater bioavailability of CAM2029 was observed compared with the group administered octreotide LAR. IGF-I concentrations were rapidly reduced and then progressively increased with the administration of all octreotide SC depot variants in a dose-dependent manner. In contrast, a slower decrease in IGF-I levels was observed after the injection of octreotide LAR, with less variation over time in hormone concentrations, but with a significant transitory increase in IGF-I levels in the first days after the second and third injections.

Of the 122 randomized participants, 105 (86.1%) completed the study—10 withdrew consent, 6 discontinued because of an AE and 1 was withdrawn after noncompliance with the protocol. During the randomized study, the most frequent AE was diarrhea (75.4%), followed by headache (49.2%). AEs were more frequently observed in the octreotide SC depot groups (86-100%) than in the octreotide LAR group (71%).

Following phase 1, a phase 2 open-label, multicenter, randomized trial was conducted that included patients with acromegaly or neuroendocrine tumors, with carcinoid syndrome being treated with octreotide LAR (10, 20, or 30 mg every 4 weeks) for at least 2 months (26). A 14-day screening period was followed by a 4-week phase, when the last dose of octreotide LAR (10, 20, or 30 mg: treatment A, B, or C, respectively) was administered. Subsequently, patients were randomized to receive 10 mg of CAM2029 every 2 weeks (acromegaly n = 3 and neuroendocrine tumors n = 1; treatment D) or 20 mg every 4 weeks (acromegaly n = 4 and neuroendocrine tumors n = 4; treatment E) for 3 months. All 12 patients who were enrolled completed the study.

The pharmacokinetics profile of CAM2029 after each administration was compared with the profile of octreotide LAR. In all cohorts, AUC_0–28_ (area under de curve between days 0–28) and *C*_max_ (maximal concentration) were considerably higher in treatments D and E than in treatments A, B, and C. IGF-I levels were also monitored and remained stable on Day 84 in 3/5 patients (60% of patients with acromegaly who had normal levels at the beginning of the study; 2/3 were in Group D and 1/3 was in Group E). The other 2 patients had IGF-I levels above normal before switching to CAM2029. Both patients continued to have high IGF-I levels, but in 1 of these patients, the levels improved. The safety profile was similar to that of octreotide LAR. The most common AEs were diarrhea (n = 3) and injection site pain (n = 3).

Subsequently, ACROINNOVA 1, a pivotal phase 3 trial (ClinicalTrials.gov number NCT04076462) investigating efficacy and safety of CAM2029 as a treatment for acromegaly using clinical, biochemical, and patient-reported outcomes, was developed (27). The study included 72 patients with controlled acromegaly previously treated with first-generation SRLs and they were randomly assigned to a double-blind treatment with either CAM2029 or placebo. The last subject study visit occurred in May 2023 and the preliminary results are expected shortly. After completing the trial, patients had the option to enter an open-label extension safety study (ClinicalTrials.gov number NCT04125836). Beyond enrolling patients completing ACROINNOVA1, the study enrolled another group of patients with active acromegaly on treatment with first-generation SRLs with different degrees of biochemical response. At this time, 135 patients were enrolled in the study. The trial is estimated to be concluded in June 2025.

## Drugs that Block the GH Receptor

Currently, there is only 1 drug that acts in the periphery, blocking the GH receptor and reducing IGF-I synthesis, pegvisomant (28). Nevertheless, other GH receptor antagonists are under development.

### Site 1-binding helix

Site 1-binding helix (S1H) is a 16-residue peptide that binds to GHR and inhibits GH-mediated STAT5 phosphorylation (29). It was designed as a direct sequence mimetic of the site 1 minihelix of human GH (residues 36-51) and mimics the minihelix contained within the “large loop” of GH site 1. This region is considered crucial for GH–GHR (growth hormone receptor) interaction.

In vitro studies showed that it is highly stable in a serum-rich environment, with more than 89% of the peptide remaining intact after 48 hours of incubation compared with 76% of pegvisomant (29). GH-mediated STAT5 phosphorylation was reduced by 67% and 36% in cell cultures of human lymphoblasts and mouse lymphoblasts, respectively (29).

The drug was also tested in a human melanoma (MALME-3 M) cell line and was able to inhibit STAT5 phosphorylation by 40% compared with 33% with pegvisomant (29). Interestingly, S1H also inhibited prolactin-induced STAT phosphorylation by 49%, which was not observed with pegvisomant, showing that S1H is an antagonist of both GH and prolactin (29, 30). Currently, there are no published studies in human subjects.

### AZP-3813

AZP-3813 is a 16 amino acid bicyclic peptide that was described as an antagonist of GHR. Previous studies have revealed that AZP-3813 was effective in rapidly decreasing IGF-I secretion until suppression in juvenile rats and in maintaining suppression when given daily for increased periods (31, 32). The suppression was directly dependent on the dose administered. In the first study, 5-week-old male Sprague Dawley rats were subcutaneously injected either with a predefined dosage of AZP-3813 (0.3, 1, 3, 10 or 30 mg/kg 2 twice a day or 10 or 30 mg/kg once a day) or with vehicle (n = 8/group) (32). Immediately before the administration of the drug and 24, 48, and 72 hours after it, blood samples were collected. A radioimmunoassay for total IGF-I was performed, revealing that 24 hours after the injection, IGF-I was already suppressed in a dose-dependent manner (highest suppression achieved with a 30 mg/kg dose either once a day or twice a day). Rats treated with AZP-3813 had a 38.4 ± 3.8% and 39.2 ± 3.7% higher decrease in IGF-I than vehicle-treated controls (10 and 30 mg/kg once a day or twice a day, respectively). There was a return of IGF-I levels to the pretreatment levels 48 hours after drug administration.

Additionally, to evaluate its effect on the maintenance of IGF-I suppression, AZP-3813 was injected subcutaneously at a daily dose of 30 mg/kg once a day or twice a day for 4 days (32). In comparison, pegvisomant was also injected in a control group daily at a dose of 100 mg/kg once a day for 4 days. Immediately before the administration of the drug on all days and subsequently 24, 48, and 72 hours after the last injection, IGF-I levels were measured. IGF-I major suppression by AZP-3813 was reached within 24 hours after the first dose (47.2 ± 2.6% decrease vs vehicle-treated controls) and was maintained through 24 hours after the last administration. In contrast, suppression by pegvisomant achieved its maximum level (32.5 ± 3.6%) 24 hours after the third dose. Therefore, AZP-3813 showed potent antagonism of GHR with a potential therapeutic effect in acromegaly.

Later, another study was performed to test the chronic effect of AZP-3813 on IGF-I suppression (31). To do so, 5-week-old male Sprague Dawley rats received SC injection of either AZP-3813 (dose 10 or 20 mg/kg once a day) or vehicle for 19 consecutive days (n = 7/group). Immediately before the first administration and subsequently on Days 1, 2, 3, 7, 10, 15, and 19, and 24 hours after the last injection, blood samples were collected. Total IGF-I was measured by radioimmunoassay. AZP-3813 was measured by liquid chromatography tandem mass spectrometry on Days 2, 10, and 19, revealing elevation of its levels from Day 2 to Day 19 by 107.1 ± 12.5 and 125.2 ± 36.3% for the 10 and 30 mg/kg doses, respectively. IGF-I was suppressed by 27.6 ± 3.4% and 29.2 ± 3.3% for the 10 and 30 mg/kg doses, respectively, after 24 hours of the first administration vs vehicle-treated control. A similar percentage of IGF-I suppression was maintained until Day 20, in contrast to the progressively increasing levels in the control group.

In the same study, femur and body length and liver weight were also analyzed: by the end of the 20 days of the study, rats treated with 10 and 30 mg/kg AZP-3813 had 4.1% and 4.6% shorter femur length, 1.1% and 3.6% shorter body length, and 7.7% and 13.3% lower liver weight, respectively, compared with vehicle-treated animals (31). By the end of the trial, rats that received the vehicle injection gained an average of 158.4 ± 4.9 g weight. In contrast, rats treated with AZP-3813 gained less weight (23.6 ± 2.1 and 27.9 ± 2.2% with doses of 10 and 30 mg/kg once a day, respectively).

More recent studies showed that AZT-3813 could act in the same manner in normal beagle dogs that received a single subcutaneous dose of 0.1, 1, or 10 mg/kg (33). The decrease in IGF-I observed 24 hours after the administration was dose related in a manner of 2.2 ± 7.1, 21.4 ± 2.7, and 27.8 ± 1.5%, respectively, for each dosage. IGF-I reduction was maintained for over 72 hours in dogs that received the highest dose. A further study used a daily dosage of 10, 30, or 60 mg/kg for 7 days (n = 2 beagle dogs/group; 1 female and 1 male) with the aim of evaluating the chronic effects of AZT-3813. All doses resulted in similar IGF-I suppression, with similar pharmacokinetic profiles after the first administration and after 7 days of treatment. Once again, the results indicate that continued treatment with AZP-3813 translates into high effectiveness in suppressing IGF-I levels in vivo in normal beagle dogs, supporting its potential role in acromegaly medical therapy.

The AZP-3813 phase 1 clinical trial study was recently started with the intent to develop a GHR antagonist as an add-on to SRL therapy in patients who do not respond properly to the primary option of medical treatment alone. Phase 1 results are expected in the first quarter of 2024.

## Conclusion

Acromegaly treatment has advanced significantly in recent decades, and the perspective is that new options will be available in a short time, helping to improve disease control and to increase treatment convenience and, therefore, improving patient quality of life. Both paltusotine and CAM2029 are tumor-directed drugs that are in more advanced stages of development (phase 3 trials) than S1H and AZP-3813 (currently only phase 1 trials) and have a higher probability of being available in the near future in clinical practice. Paltusotine will represent a novel alternative of oral treatment with a more convenient posology, and CAM2029 will allow more convenient administration of octreotide, with the possibility of self-injection. Both S1H and AZP-3813 are promising anti-GHR treatments that will potentially increase disease control, and the results of phase 2 and 3 trials are expected in the future.

## Disclosures

M.R.G. has received speaker fees from Recordati, Ipsen and Novo Nordisk, has served as a member of the advisory board of Recordati, Ipsen, Novo Nordisk, and Crinetics and as principal investigator in clinical trials from Recordati and Crinetics. M.R.G. is Associate Editor for the Journal of Clinical Endocrinology and Metabolism. L.K. has received speaker fees from Ipsen.

## Data Availability

Data sharing is not applicable to this article, as no datasets were generated or analyzed during the current study.

## Abbreviations

AE: adverse event
GH: growth hormone
GHR: growth hormone receptor
IGF-I: insulin-like growth factor type I
LAR: long-acting release
SC: subcutaneous
S1H: site 1-binding helix
SRL: somatostatin receptor ligand
SST: somatostatin receptor
ULN: upper limit of normal
